# Patient perspective and research prioritization in rehabilitation after stroke - results from an online survey in Germany

**DOI:** 10.1186/s12913-025-13824-0

**Published:** 2025-11-27

**Authors:** Torsten Rackoll, André Ketzer, Bernd Peppmeier, Anna Engel, Matthias Endres, Alexander H. Nave

**Affiliations:** 1 Department for Neurology with Experimental Neurology, Charité Universitätsmedizin Berlin, Charitéplatz 1, Berlin, Germany; 2 QUEST Center for Responsible Research, Berlin Institute of Health at Charité Universitätsmedizin Berlin, Berlin, Germany; 3 German Center for Cardiovascular Diseases, Partner Site Berlin, Berlin, Germany; 4 Stroke Alliance for Research and Development Germany (SAFED), Patient Advisory Board, Charité Universitätsmedizin Berlin, Berlin, Germany; 5 German Stroke Foundation, Gütersloh, Berlin, Germany; 6 German Center for Neurodegenerative Diseases, Partner Site Berlin, Berlin, Germany; 7 Center for Stroke Research, Universitätsmedizin Berlin, Berlin, Germany

**Keywords:** Stroke, Rehabilitation, Research priorities, Lived experience, Caregivers, Patient engagement, Germany, Survey

## Abstract

**Background:**

People with lived experience (pwle) of stroke including survivors and informal caregivers offer critical insights into research needs. This study aimed to identify stroke rehabilitation research priorities in Germany from the perspective of pwle, and to explore variation by age, sex, and time since stroke.

**Patients and methods:**

A cross-sectional, web-based survey was conducted nationwide in Germany. The survey included closed- and open-ended questions and was distributed via stroke support organizations, clinical partners, and previous study networks. Responses were analyzed descriptively.

**Results:**

A total of 470 individuals responded, including 305 stroke survivors and 133 informal caregivers. The most frequently selected research priorities were cognition and mobility. Participants aged ≤ 65 years emphasized cognition, while older participants more often prioritized mobility and speech. Women more frequently selected cognition, and men mobility. Participants with longer time since stroke emphasized secondary prevention. Open-text responses identified additional priorities, including social participation, sexuality, and the need for individualized therapy approaches. A small subgroup of caregivers of pediatric stroke survivors prioritized cognition, participation, and self-care. Overall, 75% of respondents expressed interest to be engaged in future research.

**Conclusions:**

Cognition and mobility were selected as key research priorities, with meaningful variation across demographic and clinical subgroups. These findings support the inclusion of diverse pwle perspectives in shaping national stroke research agendas and underscore the importance of tailoring rehabilitation research to stakeholder-identified needs.

**Clinical trial number:**

Not applicable.

## Background

Involving patients and other relevant stakeholders in biomedical research is widely recognized as an ethical imperative [1]. While patient and stakeholder engagement (PSE)—or patient and public involvement—has been practiced in the United States, Canada, the United Kingdom, and Australia for over two decades, active involvement in stroke research in Germany remains in its early stages [2, 3]. Broderick and Mistry recently described PSE as one of the significant advances in stroke trials [4], and the *Action Plan for Stroke in Europe* also calls for a focus on the unmet needs of people with lived experience [5]. The *Action Plan for Stroke in Europe* is an initiative taken from stroke researchers in cooperation with the *European Stroke Organisation* and the *Stroke Alliance for Europe* (SAFE) to formulate targets in stroke care to be addressed until 2030 and was first launced on 23rd of May 2018 in the European Parliament [6]. 

Unmet needs highlight the most burdensome issues faced by those with lived experience—issues that research should aim to address. Given limited resources, setting research priorities can help ensure that efforts are directed towards topics of greatest relevance to affected individuals [7]. 

Addressing the burdens and impairments associated with stroke is a global responsibility linked to the disease’s pathophysiology. However, identifying which issues are most urgent to study can be context-specific. Stroke care is delivered within diverse healthcare systems and cultural environments, which may influence the perceived importance of different research topics. Research priority setting exercises (RPSEs) have predominantly been conducted in the UK, Australia, and North America using a variety of methods — including the James Lind Alliance (JLA) methodology and Delphi approaches to stakeholder surveys [8]. Notably, the integration of patient perspectives has progressed significantly in the past decade.

The *Action Plan for Stroke in Europe* also calls for the inclusion of diverse cohorts of stroke survivors, recognising the heterogeneity in their experiences and needs [5]. For example, younger survivors aiming to return to work may face different obstacles than retired individuals, who may be more concerned with maintaining social connections. Furthermore, informal caregivers often offer unique perspectives on post-stroke impairments, adding depth to our understanding of daily life challenges—yet they remain underrepresented in RPSEs [8]. 

In a recent scoping review, we examined frameworks for patient and stakeholder engagement in stroke research [2]. To contribute to the evidence base for incorporating patient perspectives in research, we aimed to identify research priorities from a broader group of people with lived experience in Germany. To this end, we conducted a nationwide survey exploring participants’ experiences with priority and goal setting during inpatient rehabilitation, as well as their preferences regarding future stroke recovery research. Our primary objective was to identify the research topics most frequently prioritised by stroke survivors and informal caregivers. We also sought to explore whether these priorities varied by stakeholder group (survivors vs. caregivers), age group, or sex.

## Methods

We present findings from an online survey targeting stroke survivors and informal caregivers who support individuals affected by stroke. The survey received ethical approval from the local ethics committee of the Charité – Universitätsmedizin Berlin (EA 1/276/22) in accordance with the Declaration of Helsinki, and the study protocol was preregistered on the Open Science Framework (OSF) prior to data collection (https://osf.io/86k3b/). All participants provided informed consent as part of the survey process. This report adheres, where applicable, to the REPRISE reporting guideline for health research priority setting involving stakeholders [9]. However, this study was not designed to be a research priority setting exercise (RPSE) itself, but rather to provide a basis for potential future RPSE. Formal RPSE usually include the involvement of all interest holders within the research area such as clinicians, researchers or policy makers, identifying a broad range of research questions based on e.g. extensive literature review and/or stakeholder interviews, criteria by which research questions are ranked as well as some consensus exercise to agree on the ranking [10]. 

### Definitions

Terminology in PSE is not yet standardised. For this manuscript, we define the following terms:

**Table:** Definition of most relevant terms for this manuscript

Term	Definition
People with lived experience	Individuals who have personally experienced a disease or life event. In this context, it includes both stroke survivors and informal caregivers who have shared the burden of the disease alongside the survivor.
Stroke survivor	An individual who has experienced a cerebrovascular event. The term recognizes the individual’s experience rather than defining them solely by the medical event.
Informal caregiver	An unpaid individual who provides direct care to a stroke survivor and has lived through distinct disease-related experiences.
Stakeholder	Stakeholders in stroke research may include people with lived experience, clinicians, paid caregivers, policymakers, researchers, and others.

### Study design

To address the research question, we conducted an observational, cross-sectional, self-administered, online survey in German. The survey targeted individuals residing in Germany and was promoted through social media, user group newsletters (e.g., Berlin Stroke Alliance), mailing lists (e.g., German Stroke Foundation), and posters displayed at four university clinics (Charité – Universitätsmedizin Berlin, Schlossparkklinik Berlin-Charlottenburg, Universitätsmedizin Greifswald, and Ludwig-Maximilians-Universität München). In addition, participants from the Berlin-based *Rehabilitation and Clinical Observation of Stroke Patients to Validate Prognostic Factors of Functional and Subjective Recovery after Stroke* (RECOVER-S) trial (DRKS00030023) were also invited via mail if they had previously provided consent to be contacted. The survey was also advertised on the homepage of the German Stroke Society (DSG). The invitation included a brief summary of the aim of the survey as well as an access link or a QR-Code in case of the printed posters.

Due to financial limitations, the survey was only available in German. It was launched on February 13, 2025, and automatically closed after three months, on May 13, 2025.

Eligibility criteria included being 18 years or older at the time of participation of the responder and either having experienced a stroke or serving as an informal caregiver to a stroke survivor. The survey questions were developed by T.R. based on previous literature of which prominent items were selected and reviewed by individuals with lived experience of both sexes to ensure broad relevance [8, 11–14]. A representative of a stroke stakeholder group (A.E.) also reviewed the questions, and suggestions were incorporated as needed. Based on the feedback, the research area ‘Reintegration into employment’ was added and the area ‘visual impairments’ simplified which before included terms such as Neglect which was unfamiliar to some. No further adjustment to the layout of the survey was requested. The final version was reviewed and tested by researchers with experience in survey design. To enhance accessibility, we avoided abbreviations and technical terms, used lay language, and included instructions on increasing font size and using the screen reader function. The survey presented one question per screen. Open-ended questions were limited to selected items where deeper insights were necessary. The survey was implemented in *REDCap* (RRID: SCR_003445) provided by the Berlin Institute of Health at Charité Universitätsmedizin Berlin and hosted on protected servers within the university clinic infrastructure. The German codebook is available on OSF (https://osf.io/a3pt6).

Access to the survey was only granted if participants provided informed consent digitally through accepting the terms of the survey provided to them on the landing page which included the study and data protection information. After giving their consent, participants were asked to indicate whether they were stroke survivors or informal caregivers. The survey was divided into three sections: (1) experiences during inpatient rehabilitation, (2) physical activity post-stroke, and (3) research priority setting and included 18 questions in total requiring around 10–15 min to complete. This manuscript reports only on the section covering research priorities. Results related to rehabilitation experiences and physical activity will be reported separately.

### Survey questions

Table 1: Survey questions Participants could choose up to three research priorities from a predefined list (see Table 2). If “Other” was selected, a free-text option was provided for additional suggestions.

**Table 1:** Survey questions

Domain	Questions
Basic demographics	• Age • Sex • Please tell us the first two numbers of your postcode. • Have you personally suffered from a stroke? • Year of last stroke
Rehabilitation	• How many rehabilitation stays after a stroke have you had? • Please briefly state what your therapy should have focused on during your rehabilitation. • In your opinion, was the therapy plan tailored to your desired goals? • How satisfied were you with the progress you made in rehabilitation with regard to your goals?
Research prioritization and patient engagement	• What do you think research should focus on in terms of rehabilitation after a stroke? • Would you participate in research projects, for example through an advisor role or actively, if there were corresponding offers from the scientific community?

**Table 2:** Possible answers for prioritisation question

Research areas	
• Cognitive impairments • Recovery of speech • Balance • Mobility • Improvement of arm function • Secondary prevention • Visual impairments • Psychological burdens on informal caregivers • Fatigue • Fitness • Depression • Reintegration into employment • Participation in social life • Self-care and domestic life • Sexual dysfunction • Other	

Demographics were assessed to contextualize the research priorities. No compensation was provided for participation.

### Data analysis and interpretation

As is common in surveys, some data may be incomplete. Missing data were classified according to the categories described by Little and Rubin: “missing completely at random” (MCAR), “missing at random” (MAR), or “missing not at random” (MNAR) [15]. 

Two feedback rounds were conducted with members of the patient advisory board *Schlaganfall-Allianz für Forschung und Entwicklung Deutschland* (SAFED: https://safed.charite.de). Here, a summary of the results was distributed prior to the meeting with questions concerning the definition of subgroups, the cut-off for age groups, whether to report the results on underaged participants and chances to provide written comments. Further, it was discussed why open-ended fields were left blank in most cases when ‘other’ was chosen as a response. Comments were collected and used to facilitate the discussion and to guide the interpretation of the data. Feedback from these sessions was incorporated into both data interpretation and presentation which is detailed in the section ‘Patient Engagement’. A German language draft of the manuscript was shared with members of SAFED and the German Stroke Foundation to allow for additional feedback and contributions to the thematic analysis.

### Statistical analysis

Statistical analyses were performed in *R* (RRID: SCR_001905) using the *tidyverse* package (RRID: SCR_019186). All analyses were predefined.

This study aimed to describe patient and caregiver perspectives on stroke research priorities using descriptive statistics. No inferential or biometric comparisons were initially planned. The primary objective was to identify the most commonly selected research needs. The sample size was calculated to estimate proportions. Based on previous research indicating that 49% of stroke survivors emphasise the importance of studying balance and walking difficulties, a minimum sample size of 96 participants was required to achieve a 95% confidence interval with a 1% margin of error [11]. 

Results were summarized as means and percentage distributions. Comparisons between stroke survivors and informal caregivers were conducted using chi² tests for categorical, non-parametric data. Associations between age groups and responses were also analyzed.

## Results

**Fig. 1:**
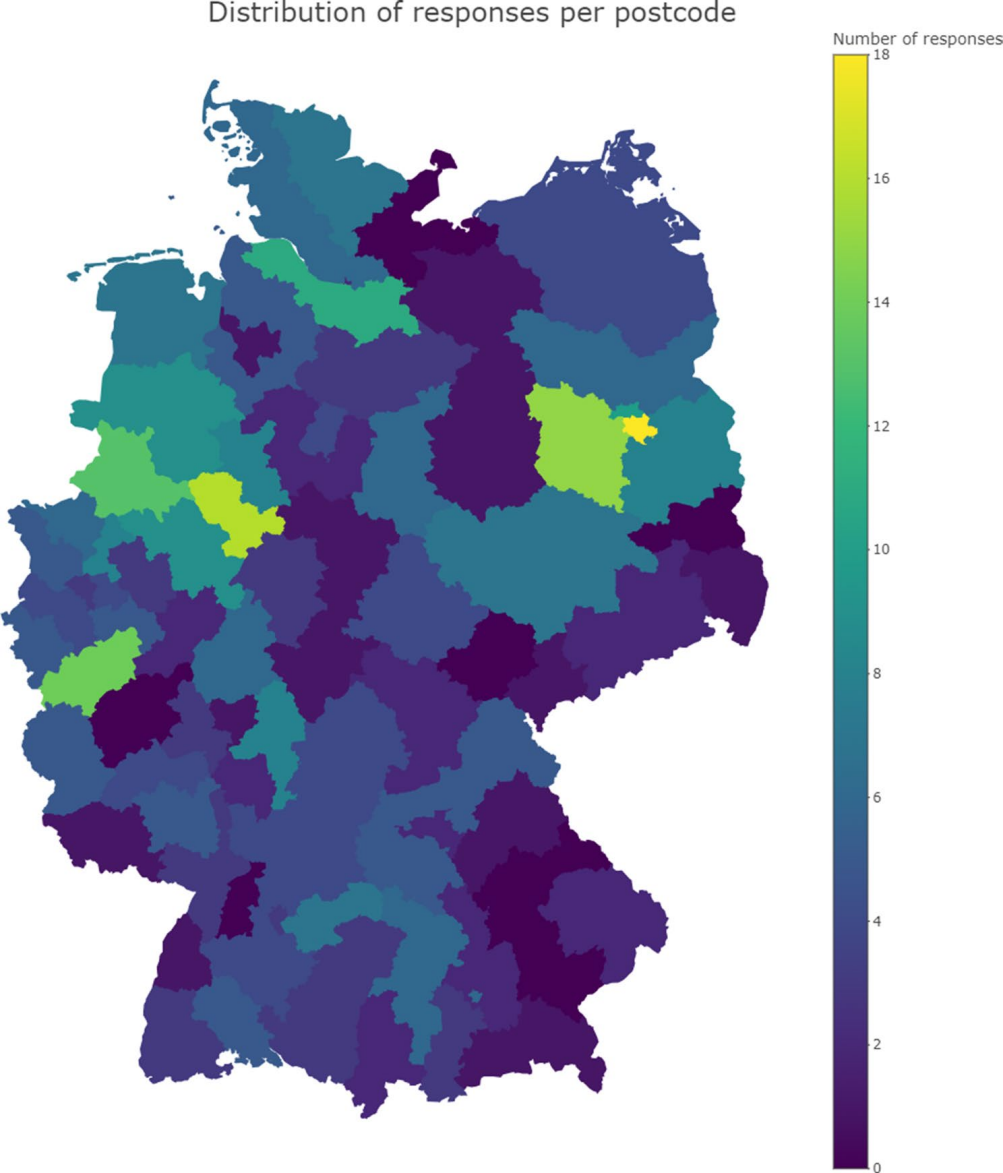
Origin of participants based on German postal codes

Within a three months period, 499 individuals accessed the survey webpage. Of these, 18 did not begin the survey and 5 declined to give informed consent. An additional 6 participants were excluded due to being under the age of 18. Consequently, 470 datasets were started and available for analysis of which 350 datasets were complete. Responses were provided from all parts of Germany (Fig. 1). Participants were able to skip questions or exit the survey at any time. Background information was provided by 438 respondents and is presented in Table 3.

The basic characteristics of participants are summarized in Table 3. Among individuals with lived experience (pwle), the majority were female, whereas a higher number of male informal caregivers responded compared to female caregivers. In both groups, strokes occurred between one to seven years prior to participation meaning that the majority of participants had more than one year of experience living with stroke.

**Table 3:** Baseline characteristics of participants

Characteristic	Informal caregiver, *N* = 133^1^	Stroke survivor, *N* = 305^1^
Age	54 (23)	53 (15)
(Missing)	3	60
Sex		
Male	79 / 132 (60%)	107 / 303 (35%)
Female	49 / 132 (37%)	195 / 303 (64%)
Diverse	2 / 132 (1.5%)	0 / 303 (0%)
No comment	2 / 132 (1.5%)	1 / 303 (0.3%)
(Missing)	1	2
Years since stroke	3 (1, 7)	4 (1, 7)
(Missing)	10	20
Number of rehabilitation stays	1 (1, 2)	1 (1, 2)
(Missing)	19	28
Was the therapy schedule tailored to your needs?		
Completely	14 / 107 (13%)	39 / 260 (15%)
Mostly	32 / 107 (30%)	83 / 260 (32%)
Partly	28 / 107 (26%)	79 / 260 (30%)
Slightly	20 / 107 (19%)	30 / 260 (12%)
Not at all	13 / 107 (12%)	29 / 260 (11%)
(Missing)	26	45
How satisfied were you with the progress you made in rehabilitation with regard to your goals		
Very unsatisfied	Not applicable	29 / 259 (11%)
Unsatisfied	Not applicable	53 / 259 (20%)
Neither nor	Not applicable	33 / 259 (13%)
Satisfied	Not applicable	109 / 259 (42%)
Very satisfied	Not applicable	35 / 259 (14%)
(Missing)	133	46

^1^Mean (SD); n / N (%); Median (IQR)

**Fig. 2:**
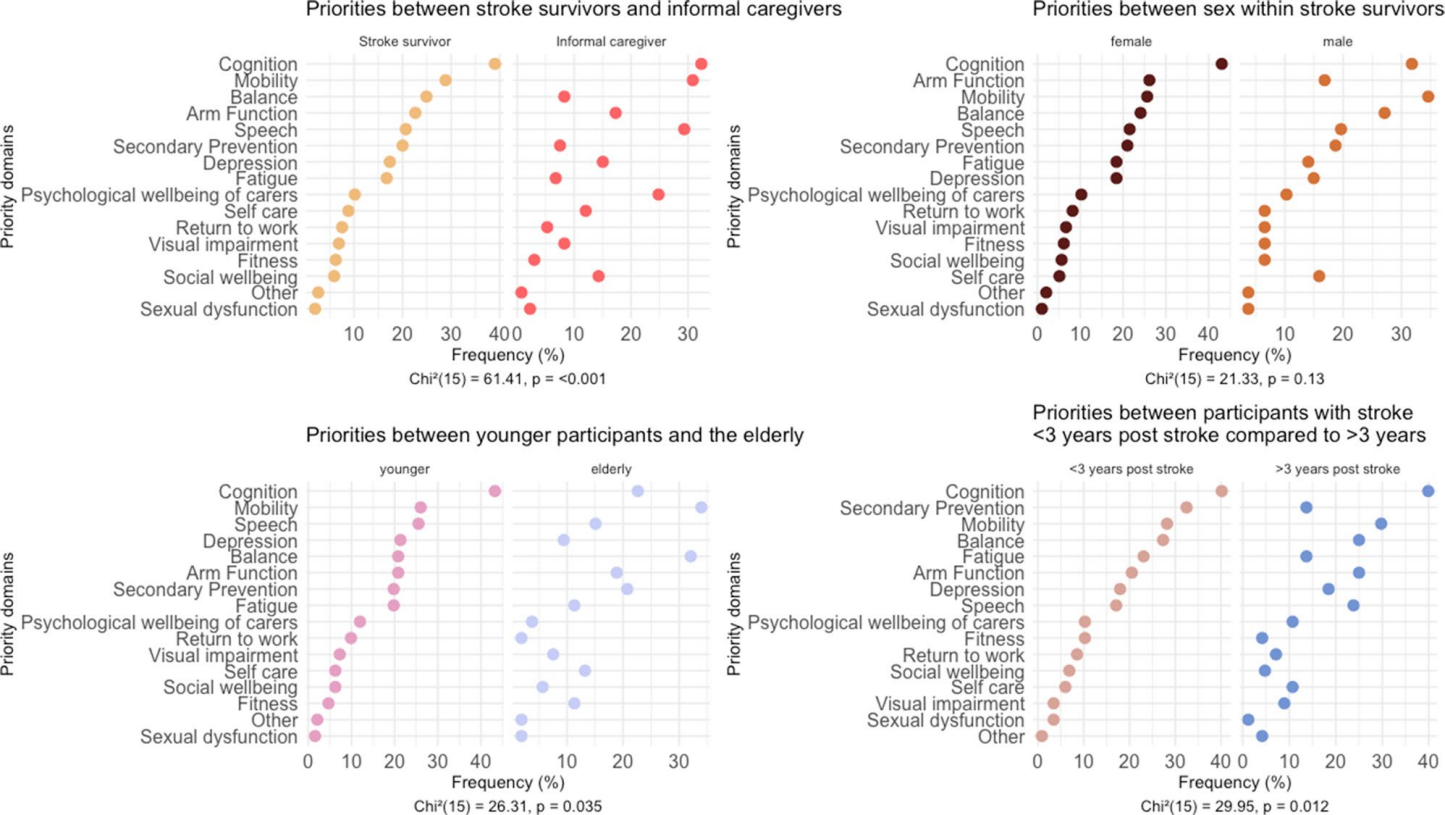
Cleveland dot plot with research priority per subgroups ordered by priority

Using predefined categories, both people with lived experience and informal caregivers were asked to identify their three most relevant research priorities. We compared responses by stakeholder group, age, and sex (Fig. 2a–c). Additionally, we examined whether research priorities depended on time since stroke, dichotomised by median value (Fig. 2d). Although participants were invited to provide additional research priorities through an open-ended question, no new topics were suggested.

We also identified a subset of 16 informal caregivers who responded on behalf of individuals under the age of 18. Due to the small sample size, no statistical comparisons were conducted between this group and older respondents. Children with stroke had a median age of 7.5 years [IQR 4–14] and a median time since stroke of 6.5 years [IQR 4.75–15]. Their reported research priorities are summarized in Table 4.

**Table 4:** Research priorities for children who had a stroke, ranked by number of votes

Priority	Votes (*n* = 16)
Cognition	7
Participation	4
Arm function	4
Self-care	4
Psychological wellbeing of carers	3
Speech	2
Balance	1
Fatigue	1
Fitness	1

Finally, we asked participants whether they would be willing to take part in a patient and stakeholder engagement (PSE) framework if such a structure was available in stroke research. Of the 470 participants, 345 responded to this question. The majority (*n* = 258 expressed interest in participating, while 24 indicated they would not, and 63 responded ‘maybe’. Participants in the “maybe” group were given the opportunity to elaborate on their response through an open-ended comment field. The majority of these comments referred to potential logistical barriers or time constraints. Illustrative quotes include:

“The projects must fit into a time frame, possibly within a short travel distance.” — Person with lived experience.

“Online surveys” — Person with lived experience.

Some participants also mentioned enabling factors:

“My psychological situation” — Person with lived experience.

### Patient engagement

Pwle were involved throughout the study process. Their feedback informed adjustments to survey language and the inclusion of additional predefined research priority categories. Findings were discussed in two consultation rounds (24. June 2024 & 23. September 2024), during which several insights emerged:

First, participants emphasized the importance of stratifying data by age. A cutoff at age 65 was considered particularly relevant within the German context, where retirement age influences coverage by different insurances and access to rehabilitation services. Pediatric stroke was viewed as a distinct category requiring separate consideration.

Second, the predefined answer options were considered too broad. While “cognition” was most frequently selected, it did not fully capture the range of individual cognitive challenges. Participants mentioned subtle but impactful challenges — such as a lingering sensation described as “brain fog” — that impact quality of life but are difficult to articulate. Others expressed a desire to see underrepresented areas, such as sexuality and intimate relationships, reflected in research priorities. These perspectives highlight a tension between individual needs and generalized, group-based therapy models. The need for truly personalized rehabilitation approaches was strongly emphasized.

Third, participants highlighted logistical feasibility as a prerequisite for engagement in research. Suggestions included more flexible and accessible formats, such as home visits and telehealth options. Several noted that incomplete open text responses may reflect emotional fatigue or difficulty of revisiting personal impairments — a reminder of the sensitive nature of the subject matter.

## Discussion

In this study, we assessed research priorities among stroke survivors and informal caregivers in Germany using an online survey. To our knowledge, this is the first study in Germany to explore stroke recovery research priorities from these stakeholder perspectives [8]. While similar studies have been conducted internationally, country-specific investigations are necessary to account for differences in healthcare systems, service delivery, and local community contexts. Such specificity enables alignment of publicly funded research with the real-world needs of its beneficiaries.

Previous research has often selected participants based on stroke severity or through convenience sampling from prior studies [11, 12]. In contrast, we aimed to capture a broad and diverse spectrum of perspectives by employing multiple distribution channels. These included scientific and patient representative organizations, posters in clinical settings, and postal invitations to former study participants. As a result, our sample includes responses from across all regions of Germany.

Cognitive impairments were prioritized most highly by both stroke survivors and informal caregivers. This finding aligns with the result of the most robust stroke research priority-setting initiative to date: the James Lind Alliance (JLA) Stroke Priority Setting Partnership [13]. In its initial exercise, the question *“What are the best ways to improve understanding (cognition) after stroke?”* emerged as a top priority. The updated 2021 survey reinforced cognition and psychological wellbeing as dominant themes in rehabilitation and long-term care [16].

Our subgroup analyses revealed age-related differences. While cognition remained the top priority for participants aged ≤ 65, older participants gave higher priority to mobility and speech impairments. Rudberg et al. similarly reported age-related differences: respondents ≥ 70 years prioritized balance and mobility, while younger participants emphasized speech and fatigue [11]. In our sample of underaged pwle, cognition, participation, and self-care emerged as key concerns. These findings echo those of Gordon et al., who noted that pediatric stroke uniquely affects daily life and social participation [17]. This suggests the need for distinct assessment and intervention frameworks for children.

We also found sex-based differences: women prioritized cognition, whereas men more often emphasised mobility. Although few comparable studies have analysed sex-specific preferences, recent literature points to gender-based disparities in stroke outcomes and care [18]. Our study does not provide sufficient data to explain these differences but underscores the importance of future investigation.

Time since stroke also influenced research priorities. Although cognition remained a consistent concern regardless of time elapsed, participants further removed from their stroke event were more likely to prioritize secondary prevention. This may reflect a shift from acute recovery needs to long-term management challenges. Supporting this interpretation, Franzisket et al. recently reported that pwle in Germany cite persistent issues with memory, concentration, and access to continued rehabilitation [19]. Of note, most participants expressed at least one year of experience living with stroke meaning that our results are more directed to chronic patients. Previous studies indicate a temporal shift in priorities after stroke, with early stages characterized by emotional adjustment and coping with loss of function, and later stages emphasizing maintenance of physical function and secondary prevention [20, 21]. 

In feedback rounds, participants highlighted nuanced cognitive symptoms such as “brain fog.” This term, frequently discussed in relation to long COVID, is described by the American Stroke Association as “a range of different symptoms that can make you feel like you’re not thinking clearly [22]. The importance of sexual health was also emphasized, consistent with recent evidence reporting a 14–50% prevalence of sexual dysfunction in the early months following stroke [23].

## Limitations

This study used an online survey, inherently limiting participation to those with digital access and literacy. Although we incorporated accessibility features (e.g., adjustable font size, text-to-speech), individuals with cognitive, visual, or language impairments—such as aphasia—may still have been unable to participate. Our approach included multiple outreach strategies, but we lacked resources for postal surveys, which may have excluded further segments of the stroke population. In addition, the survey was a self-administered online questionnaire. We had no possibility to verify the responses that were provided.

The predefined priority categories may have constrained participant responses, even though open-text fields were available. The broad framing of categories was intentional to initiate a national conversation about research needs. This study is not a substitute for a formal research priority setting (RPS) initiative, such as those following the James Lind Alliance methodology or Cochrane Priority Setting frameworks [24, 25]. Rather, it provides a foundational understanding of current stakeholder views, which should be expanded through future structured exercises that also include clinicians and researchers.

### Future directions

Following the survey, a national advisory group was established — SAFED (Schlaganfall Allianz für Forschung und Entwicklung Deutschland) — to support patient and stakeholder involvement in stroke research. A formal RPS process is recommended as the next step, aligned with REPRISE guidelines and Cochrane methods [24, 25]. Such a process should draw on the current findings and ensure that future projects, particularly those focused on cognition and mobility, include pwle as active partners throughout the research lifecycle.

## Conclusion

This study identified cognition and mobility as top research priorities for stroke recovery among stroke survivors and informal caregivers in Germany. Subgroup analyses revealed differences by age, sex, and time since stroke, highlighting the need for tailored approaches. Future research should engage pwle at every stage to ensure that research remains relevant, equitable, and impactful.

## Data Availability

The code to produce this manuscript including the analysis scripts are shared in a public online github repository (https://github.com/Funkstille1011/PRIO-Stroke). Protocol and processed data are available on our open science framework (osf) repository ([https://osf.io/86k3b/] ([10.1186/s13643-024-02686-y)).

## References

[1] Arumugam A, Phillips LR, Moore A, et al. Patient and public involvement in research: a review of practical resources for young investigators. BMC Rheumatol. 2023;7:2. 36895053 10.1186/s41927-023-00327-w PMC9996937

[2] Khankeh H, Guyatt G, Shirozhan S, et al. Stroke patient and stakeholder engagement (SPSE): concepts, definitions, models, implementation strategies, indicators, and frameworksa systematic scoping review. Syst Reviews. 2024;13:271. 10.1186/s13643-024-02686-y PMC11526530 39482702

[3] Pfisterer-Heise S, Iannizzi C, Messer S, et al. Stakeholders’ perspectives on patient involvement in systematic reviews results of a world Café in Germany. Zeitschrift für Evidenz Fortbildung Und Qualität Im Gesundheitswesen. 2024;188:26–34. 39043520 10.1016/j.zefq.2024.06.003

[4] Broderick JP, Mistry EA. Evolution and future of stroke trials. Stroke. 2024;55:1932–9. 38328974 10.1161/STROKEAHA.123.044265 PMC11196204

[5] Norrving B, Barrick J, Davalos A, et al. Action plan for stroke in Europe 20182030. Eur Stroke J. 2018;3:309–36. 31236480 10.1177/2396987318808719 PMC6571507

[6] Stroke action plan for Europe (SAP-E). Stroke action plan for Europe. Accessed 11.06.2025. https://actionplan.eso-stroke.org/

[7] Chalmers I, Bracken MB, Djulbegovic B, et al. How to increase value and reduce waste when research priorities are set. Lancet. 2014;383:156–65. 24411644 10.1016/S0140-6736(13)62229-1

[8] Leitch S, Logan M, Beishon L, et al. International research priority setting exercises in stroke: A systematic review. Int J Stroke. 2023;18:133–43. 35422174 10.1177/17474930221096935 PMC13020986

[9] Tong A, Synnot A, Crowe S, et al. Reporting guideline for priority setting of health research (REPRISE). BMC Med Res Methodol. 2019;19:243. 31883517 10.1186/s12874-019-0889-3 PMC6935471

[10] Nasser M, Viergever RF, Martin J. Prioritization of research. In: Kayona R, Clarke M, Murray V, et al. editors. WHO guidance on research methods for health emergency and disaster risk Management, revised 2022. Geneva: World Health Organization; 2022. pp. 122–34.

[11] Rudberg A-S, Berge E, Laska A-C, et al. Stroke survivors’ priorities for research related to life after stroke. Top Stroke Rehabil. 2021;28:153–8. 32627722 10.1080/10749357.2020.1789829

[12] Turner GM, Backman R, McMullan C, et al. Establishing research priorities relating to the long-term impact of TIA and minor stroke through stakeholder-centred consensus. Res Involv Engagem. 2018;4:2. 29416879 10.1186/s40900-018-0089-z PMC5784709

[13] Pollock A, St George B, Fenton M, et al. Top 10 research priorities relating to life after stroke consensus from stroke Survivors, Caregivers, and health professionals. Int J Stroke. 2014;9:313–20. 23227818 10.1111/j.1747-4949.2012.00942.x

[14] Sangvatanakul P, Hillege S, Lalor E, et al. Setting stroke research priorities: the consumer perspective. J Vasc Nurs. 2010;28(4):121–31. 21074114 10.1016/j.jvn.2010.09.001

[15] Little RJ, Rubin DB. Statistical analysis with missing data, 2nd Edition | Wiley. Wiley. (2014). https://www.wiley.com/en-it/Statistical+Analysis+with+Missing+Data%2C+2nd+Edition-p-9781119013563

[16] Hill G, Regan S, Francis R. Stroke Priority Setting Partnership Steering Group. Research priorities to improve stroke outcomes. Lancet Neurol. 2022;21(4):312–3. 10.1016/S1474-4422(22)00044-8 PMC8926410 35305334

[17] Gordon AL, Nguyen L, Panton A, et al. Self-reported needs after pediatric stroke. Eur J Pediatr Neurol. 2018;22:791–6. 10.1016/j.ejpn.2018.06.003 29960841

[18] Walter S, Sacco S, Sandset EC. Women and leadership in stroke clinical trials: time for a call to action. Stroke. 2023;54:304–5. 36300373 10.1161/STROKEAHA.122.041227

[19] Franzisket C, Voigt C, Brinkmeier M, et al. Unerfüllte Bedürfnisse in der Schlaganfall-Nachsorge auswertung einer befragung von Schlaganfall-Betroffenen in Deutschland. Das Gesundheitswesen. Epub ahead of print 13 March 2025. 10.1055/a-2525-2857 10.1055/a-2525-2857 40081410

[20] Hughes AK, Cummings CE. Grief and loss associated with stroke recovery: A qualitative study of stroke survivors and their spousal caregivers. J Patient Exp. 2020;7(6):1219–26. 33457568 10.1177/2374373520967796 PMC7786670

[21] Lo SHS, Chau JPC, Lam SKY, et al. Understanding the priorities in life beyond the first year after stroke: qualitative findings and non-participant observations of stroke survivors and service providers. Neuropsychol Rehabil. 2023;33(5):794–820. 35261329 10.1080/09602011.2022.2049827

[22] American Stroke Association. Brain Fog. Accessed: 12.11.2025. https://www.stroke.org.uk/stroke/effects/physical/brain-fog

[23] Dusenbury W, Barnason S, Vaughn S, et al. Sexual health after a stroke: A topical review and recommendations for health care professionals. Stroke. 2025;56(5):1312–22. 40116003 10.1161/STROKEAHA.124.044723

[24] James Lind Alliance. The james lind alliance guidebook (version 10). https://www.jla.nihr.ac.uk/jla-guidebook

[25] Nasser M, Welch V, Ueffing E, et al. Evidence in agenda setting: new directions for the Cochrane collaboration. J Clin Epidemiol. 2013;66:469–71. 23312393 10.1016/j.jclinepi.2012.08.006

